# IS*4 *family goes genomic

**DOI:** 10.1186/1471-2148-8-18

**Published:** 2008-01-23

**Authors:** Daniel De Palmenaer, Patricia Siguier, Jacques Mahillon

**Affiliations:** 1 Laboratoire de microbiologie alimentaire et environnementale, Université catholique de Louvain, Croix du Sud 2/12, B-1348 Louvain-la-Neuve, Belgium; 2 Laboratoire de microbiologie et génétique moléculaires, Université Paul Sabatier, 118 route de Narbonne, 31062 Toulouse cedex 9, France

## Abstract

**Background:**

Insertion sequences (ISs) are small, mobile DNA entities able to expand in prokaryotic genomes and trigger important rearrangements. To understand their role in evolution, accurate IS taxonomy is essential. The IS*4 *family is composed of ~70 elements and, like some other families, displays extremely elevated levels of internal divergence impeding its classification. The increasing availability of complete genome sequences provides a valuable source for the discovery of additional IS*4 *elements. In this study, this genomic database was used to update the structural and functional definition of the IS*4 *family.

**Results:**

A total of 227 IS*4*-related sequences were collected among more than 500 sequenced bacterial and archaeal genomes, representing more than a three fold increase of the initial inventory. A clear division into seven coherent subgroups was discovered as well as three emerging families, which displayed distinct structural and functional properties. The IS*4 *family was sporadically present in 17 % of analyzed genomes, with most of them displaying single or a small number of IS*4 *elements. Significant expansions were detected only in some pathogens as well as among certain extremophiles, suggesting the probable involvement of some elements in bacterial and archaeal adaptation and/or evolution. Finally, it should be noted that some IS*4 *subgroups and two emerging families occurred preferentially in specific phyla or exclusively inside a specific genus.

**Conclusion:**

The present taxonomic update of IS*4 *and emerging families will facilitate the classification of future elements as they arise from ongoing genome sequencing. Their narrow genomic impact and the existence of both IS-poor and IS-rich thriving prokaryotes suggested that these families, and probably ISs in general, are occasionally used as a tool for genome flexibility and evolution, rather than just representing self sustaining DNA entities.

## Background

Insertion sequences (ISs) are small (< 2.5 kb), generally phenotypically cryptic segments of DNA able to jump, or copy themselves, into various genomic sites with no need for DNA homology [1]. They generally encode no functions other than those involved in their mobility, although elements including additional genes are now being identified [2,3]. While almost exclusively restricted to bacterial and archaeal genomes, they are, like eukaryotic transposable elements, involved in a wide variety of biological transactions leading to genome reshuffling and evolution. Indeed, their ability to proliferate within a genome provides the potential for homologous recombination-mediated deletions or inversions, and their capacity to transport accessory genes represents an additional contribution to genome flexibility. By affecting gene expression and facilitating the emergence of new gene clusters they play an important role in adaptability of their host. Eventually, horizontal transfer mechanisms such as conjugation allow these IS-mediated sets of genes to cross barriers between strains, species and beyond [4].

The DNA breaks and joins necessary for transposition are catalysed by an element encoded protein referred to as transposase. These proteins determine transposition mechanisms and are now used to lead classification of prokaryotic transposable elements in general. Accordingly, transposases that form a covalent intermediate with DNA are distinguished from those that do not. Additional distinction is provided by protein active-site residues crucial for transposition. These define the five major transposase classes currently established : tyrosine (Y), serine (S), relaxase (Y1) and rolling-circle (Y2) transposases involve covalent intermediates with DNA during transposition, while the fifth class, namely DDE transposases, prompts transposition via direct transesterification reactions [5-7].

DDE transposases display three acidic residues in three distinct regions of their primary sequence, namely regions N2, N3 and C1, which harbor the aspartate (D), aspartate (D) and glutamate (E) residues, respectively. Spacers of various lengths separate these regions, but their acidic residues are brought together upon protein folding to form a catalytic triad essential for transposition [8-11].

Prokaryotic DDE transposons (mainly ISs) can move in two different ways, depending on the fate of the donor site. Replicative transposons mobilize a copy of their DNA, leaving the parent site intact, while conservative transposons cut themselves out of the donor molecule in order to paste their DNA into the target [12].

Beyond mechanistic behaviors, each individual IS is characterized by structural features used to fine-tune their classification. A wealth of these data is currently generated with the rising availability of whole genome sequencing projects. As of July 2007, more than 19 different IS families are established based on over 1800 bacterial and archaeal IS sequences [1,13-16]. An IS family can be defined as a collection of elements sharing the same catalytic site structure (with conserved spacers between key residues), an identical genetic organization (e.g. frameshifting in transposase gene), similar arrangements of their ends and uniform target site fates upon insertion. However, not all families are so coherent. This is why some of them (like families IS*4 *and IS*5*) are divided into subgroups being composed of a core of closely related elements that can be linked to other members of the family by weaker but still significant similarities.

The IS*4 *family, like most IS families described so far, contains elements mobilized by DDE transposases performing a 'cut-and-paste' mechanism. The main and almost only hallmarks of this family's transposases are (i) absence of frameshift in the transposase gene and (ii) an Y-(2)-R-(3)-E-(6)-(K) signature (YREK) in region C1 where the glutamate residue is that of the DDE motif [1,17]. Only very few IS*4 *elements have been studied in detail. IS*231A *has been shown to transpose *in vivo *by a 'cut-and-paste' mechanism [18], both in its natural host, *Bacillus thuringiensis *[19], and in *Escherichia coli *[20]; and it displays a certain degree of insertion specificity [21]. Both IS*10R *and IS*50R *are part of composite transposons (Tn*10 *[22,23] and Tn*5 *[24,25], respectively) and are the only members for which *in vitro *systems have been set up. The latter has allowed extensive acquisition of genetic, biochemical, mechanistic and regulation data for these elements. Also, the Tn*5 *transposase is the only IS*4 *element for which X-ray crystallographic structure data are available [9,26].

Since the initial definition of family IS*4 *(based on about 45 elements), other elements were progressively added via ISfinder, the prokaryotic IS database [15]. However, some of them displayed distant resemblance both to existing IS*4 *members as well as members of other groups such as the IS*5 *family or ISNCY (IS Not Classified Yet). Some elements even lacked the above mentioned distinguishing protein motifs. It was therefore necessary to perform a systematic screen for related elements in order to gain a more rational view of the organization of the IS*4 *family.

Here we report an extensive *in silico *search for IS elements related to family IS*4 *among more than 500 complete bacterial and archaeal genomes. A total of 227 putative intact IS*4*-related elements were collected and permitted a detailed update of the IS*4 *catalogue, together with the description of novel emerging IS families. This allowed the evaluation of their distribution and impact among major prokaryotic phyla. Finally, known transposition mechanisms could be discussed in light of novel primary sequence data.

## Results

### Classification process

**Figure 1:**
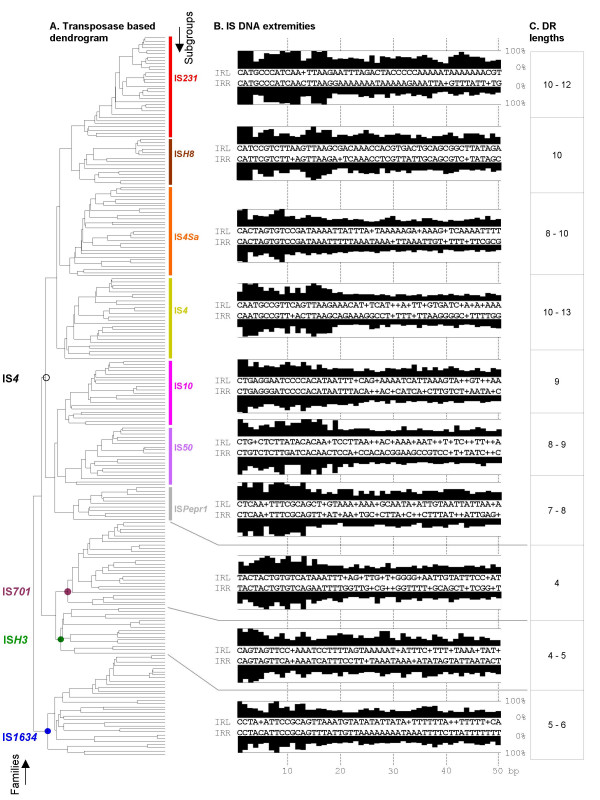
**Transposases *vs*. IRs and DRs**. Comparative overview of IS sequence features. **A. **Dendrogram representing an alignment of 227 transposases. The first common node of each family was pointed out on the left tree side. Subgroups of family IS*4 *are mentioned on the right tree side. Individual IS names were removed for clarity. **B. **Consensus sequences (5' to 3') of left (upper line) and right (lower line) DNA extremities of distinct subgroups/families. '+' symbols are used when the highest conservation level is shared by more than one residue. Percentages of nucleotide conservation at all positions are indicated by black bars. Decimal nucleotide numbering is marked by dotted vertical lines. Full alignments that generated each consensus can be found in Additional files 1 to 10. Note that in the case of family IS*701*, the exposed consensus does not represent the entire family. For further details, see Additional file 8. IRL, left TIR; IRR, right TIR. **C. **Target duplication length range in bps.

The present assignment of families and subgroups is primarily based on transposase and DNA end sequence data. Analysis of transposases was performed by multiple sequence alignments and clustering methods followed by dendrogram construction to set up clusters of related proteins (see Methods). Left and right DNA extremities flanking transposase genes were aligned (i) to each other to facilitate observation of terminal inverted repeats (TIRs) and (ii) with TIRs from other ISs to detect DNA extremity conservations. Together, these approaches split the initial IS set into ten groups. In each of them, the percentage of residue identity among transposase pairs often varied between 20 % and 50 %, highlighting the magnitude of divergence occurring among elements of a same IS group. The length of TIRs ranged from 10 to 40 bp and many of them were imperfect. Comparison of TIRs from different elements of a given IS group uncovered unique and conserved signatures in each of them, illustrating the relationship between the transposase sequence and the IS terminal repeats (Figure 1).

**Figure 2:**
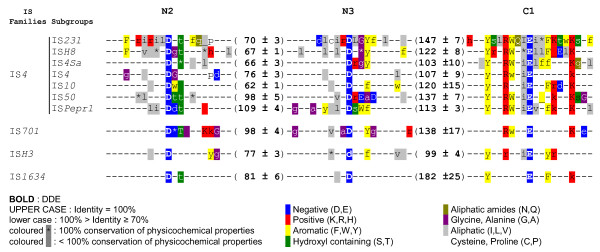
**DDE and YREK motifs of IS*4 *subgroups and emerging families**. Comparative overview of conserved transposase regions among IS*4 *subgroups and emerging families. Each line represents a part of the amino acid consensus obtained from multiple alignments of members belonging to the displayed IS groups. Numbers in brackets correspond to the mean amino acid spacer (accompanied by standard deviation) between the two aspartate residues or the aspartate and glutamate residues of the DDE motifs among transposases form a given group. The conserved transposase regions N2, N3 and C1 are mentioned on top of alignment. Symbols and colors are used as depicted in keys.

**Figure 3:**
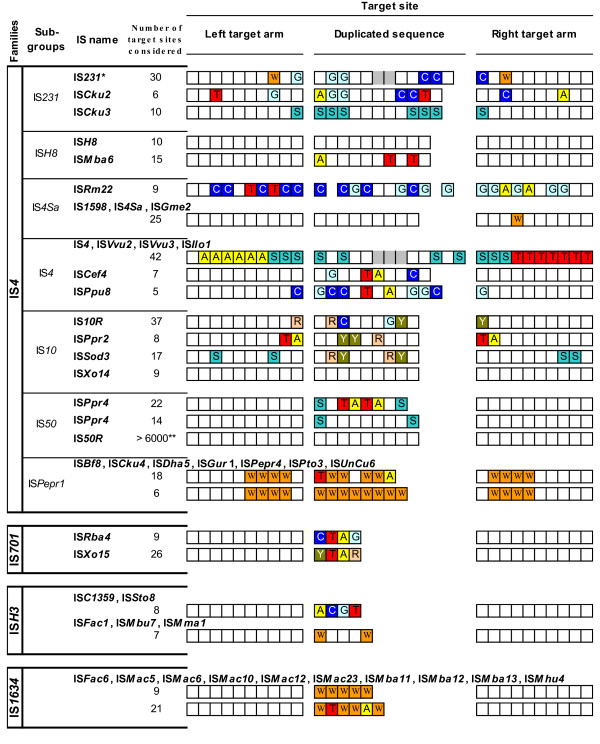
**Target sites : duplications and conservations**. Consensus sequences of *in silico *reconstructed and aligned target sites (5' to 3') typical for each subgroup or family. Only ISs found at least at five different genomic locations and flanked by DRs were considered, as well as elements described previously in literature. Each box represents one base position. Target sites are divided in three parts. The central sequence, which is duplicated upon insertion, is flanked by one upstream (left) and one downstream (right) target arm. Ten bps of each arm are shown. The number of target sites considered for each element is indicated. If more than one element displayed similar insertion specificity, their insertion sites were combined into a single line and their names listed above it. Gray boxes inside a duplication consensus indicate that DRs of variable length can be found for the given element. W, A or T; S, G or C; R, A or G; Y, T or C. * 'IS*231*' stands for following elements: IS*231A*, *C*, *F*, *M*, *S*, *T*, *U*, *Y*, IS*Bce4*, *5*, *6*, *11*, *12 *and IS*Bth5*. ** For the 6000 sites, see references [75–77].

To validate this clustering, established groups were analysed independently for conservation of specific transposase domains as well as length and specificity of target site duplications. Analysis of transposase DDE catalytic regions (N2, N3 and C1) revealed a perfect conservation of the DDE motif (Figure 2), while the size and sequence of the spacers that separate these catalytic residues differed among the ten groups. The YREK motif was partially lacking in three groups, where either its tyrosine, arginine or lysine residue was not conserved. According to the definition of IS families, IS groups sharing the same catalytic site structure were assigned together. Seven clusters displayed the complete YREK motif; and were named subgroups IS*231*, IS*H8*, IS*4Sa*, IS*4*, IS*Pepr1*, IS*10 *and IS*50*. The three remaining groups all displayed a distinct variation of the YREK motif and were therefore assigned to different and new families, namely IS*701*, IS*H3 *and IS*1634*, referred as emerging families. Finally, while IS*4 *family members generated about 10 bp target duplications, i.e. the approximate length of a complete DNA helix turn, those from emerging families displayed direct repeats (DRs) of around five bps, which corresponds approximately to half a DNA helix turn (Figures 1 &3).

### Review of family IS*4*

Family IS*4 *included 153 distinct intact elements. The main hallmarks were the presence of D(60~110)D(100~150)E and Y(2)R(3)E(6)K motifs, single *orf *encoded transposases and target site duplication lengths corresponding approximately to one DNA helix turn. Considerable diversity was observed at the level of transposase regions (sequence and length) outside catalytic residues, DNA end signatures and target site specificity. The following description of established subgroups will summarize this diversity.

**Figure 4:**
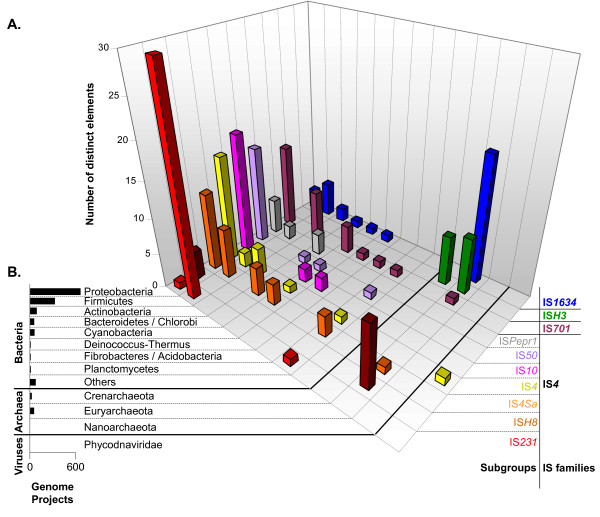
**Prokaryote distribution of IS*4 *subgroups and emerging families**. **A. **Three dimensional histogram of the number of distinct elements belonging to IS*4 *subgroups and emerging families, as they can be found among major prokaryotic clades. Each IS subgroup/family is represented by a different color. Iso-forms (which we defined as elements that show a divergence of less than 2% in the amino acid sequence of their potential proteins) were not included. The line 'Others' stands for Aquificae, Chlamydiae/Verrucomicrobia, Chloroflexi, Chrysiogenetes, Dictyoglomi, Fusobacteria, Nitrospirae, Spirochetes, Thermodesulfobacteria and Thermotogae. For interpretation, see main text. **B. **To avoid misinterpretation triggered by unequal sequencing efforts between different microbial groups, the number of genome projects, as of 1^st ^April 2007, is depicted by histogram.

**Subgroup IS*231 ***was previously established (reviewed in reference [2]) and was the most coherent one since many transposase pairs displayed sequence identity percentages above 50 (see also Figure 1A). Likewise, their DNA ends are among the most conserved. A clear relationship was observed between transposase and extremity conservation (Additional file 1). This is particularly true for left extremities (conventionally upstream of the transposase gene) where transposase-related conservation extended beyond the TIRs. The IS*231 *subgroup further distinguished itself by the fact that its members occurred almost exclusively in genomes of phylogenetically close bacteria composing the *B. cereus sensu lato *group (i.e. *B. cereus sensu stricto*, *B. thuringiensis *and *B. anthracis*, see Additional file 1 and Figure 4). As previously shown, an important hallmark is the large size range of its elements (Table 1). A significant fraction of them displayed additional DNA between the left TIR and the transposase gene [2,3], which is still unusual among ISs. In this study, novel putative passenger genes were found in these extra sequences coding for resistance, virulence or metabolism determinants as well as unknown *orf*s (results not shown). This modular aspect has not yet been observed in other IS families or IS*4 *subgroups, which may be due to the fact that, so far, no systematic and thorough searches for supplementary IS DNA were carried out in other families.

**Table 1:** Major features of IS*4 *subgroups and newly defined families

Families	Sub-groups	Typical size range (bp)	Ends^*a*^	YREK^*b*^	Direct repeat length (bp)
IS*4*	IS*231*	1450 – 5400	CAT-------AA--TAA---	Y	10 – 12
	IS*H8*	1400 – 1800	CAT-----------T-----	Y	10
	IS*4Sa*	1150 – 1750	CA------T-----------	Y	8 – 10
	IS*4*	1400 – 1650	-AATR--------WTW----	Y	10 – 13
	IS*10*	1200 – 1350	CT------------------	Y	9
	IS*50*	1350 – 1550	CW------Y---CA-A--W-	Y	8 – 9
	IS*Pepr1*	1500 – 1600	YT-AA-YTT---A-------	Y	7 – 8
IS*701*	-	1400 – 1550	---YACT-Y--YR-------	N	4
IS*H3*	-	1225 – 1500	CRGT----------------	N	4 – 5
IS*1634*	-	1500 – 2000	C-----YT------------	N	5 – 6

^a^Conserved terminal based pairs. Capital letters refer to mostly conserved bases. W, A or T; R, G or A; Y, T or C.

^b^Presence (Y) or absence (N) of YREK motif.

The new organization of family IS*4 *is shown together with emerging families. The table summarizes DNA characteristics with transposase motifs and target site duplication lengths. The two major differences between IS*4 *and emerging families are presence or absence of the YREK motif and dissimilar lengths of DRs.

**Subgroup IS*H8 ***was initially included into subgroup IS*4Sa *[1]. Yet, our results showed a closer proximity to subgroup IS*231*; not only at the transposase level, but also due to DNA extremity conservations (both displayed conserved 5'-CAT ends, Figure 1B and Additional file 2). IS*H8 *elements were initially discovered in archaea but recent genome projects uncovered elements of this subgroup in proteobacteria. Examination of IS*H8 *insertions did not reveal apparent target site specificity, contrary to the one observed for IS*231 *elements (Figure 3).

#### **Subgroups IS*4Sa *and IS*4***

This study provided the first thorough description of subgroup IS*4Sa *that was originally mentioned in reference [1]. Together with IS*4Sa*-like elements appeared a closely related subgroup including element IS*4*, which provided the name for this second ensemble. Subgroups IS*4Sa *and IS*4 *were relatively close at the protein level and the major argument for splitting them in two was a different organization of TIRs (Figure 1B and Additional files 3 &4). Extremities in subgroups IS*4Sa *and IS*4 *displayed relaxed conservation of the third and first nucleotide, respectively, which is rather new but not unique in family IS*4 *(see below). Some elements of subgroup IS*4 *displayed spectacular apparent insertion specificity in left and right target arms, a conservation that was also observed for IS*Rm22 *from subgroup IS*4Sa *(Figure 3). Both subgroups were quite diverse and broadly distributed in bacteria. Rare occurrences were recorded in archaea (IS*Fac10*) and algae viruses (ISv*EsV1_1*, see Figure 4 and Additional files 3 and 4).

**Subgroups IS*10 *and IS*50 ***were the only ones that did not cluster together with subgroups IS*231*, IS*H8*, IS*4Sa*, IS*4 *and IS*Pepr1 *during Tribe-MCL analysis (results not shown), indicating more distant relationships with these subgroups. This was also observed at the level of DNA ends since they systematically displayed 5'-NT extremities instead of 5'-NA. Yet, they displayed key residue conservation (Figure 2) and target site duplication lengths comparable to typical IS*4 *elements (Figures 1 &3). So far, the majority of both subgroups occurred among proteobacteria.

**Subgroup IS*Pepr1 ***was also newly established here and is almost exclusively composed of novel elements. Although it is composed of a limited repertoire, it is already forming a consistent subgroup with apparent insertion preference for AT-rich sequences (Figure 3) and, like elements of subgroups IS*10 *and IS*50*, it preferentially displays 5'-CT ends (Additional files 5, 6 and 7), in contrast to 5'-CA extremities encountered in the other elements from family IS*4*.

### Emerging families

Members of **Family IS*701 ***were already considered distantly related to IS*4 *in reference [1] and this was confirmed here. Only four of the 27 considered IS*701 *elements displayed the tyrosine of the YREK motif (results not shown, see Figure 2); and almost all elements displayed a highly conserved target site duplication of exactly four bps (results not shown, see Figure 3). The diversity emanating from IS*701 *transposases and TIRs allowed identification of three distinct clusters (Additional file 8), which announced a possible division into subgroups. As indicated by their prokaryotic distribution, family IS*701 *seemed to be rather widespread (Figure 4).

**Family IS*H3 ***was a small group so far restricted to archaea (Additional file 9). Half of their transposases lacked the lysine residue of the YREK motif while all (except IS*Fac10*) displayed a Y-(2)-R-(3)-E-(3)-(R) motif. DRs flanking IS*H3*-like insertions are typically five bps long and generally flanked by A at one end and T at the other end.

**Family IS*1634 ***was initially named IS*1549 *[1]. This new designation should prevent confusion with an emerging group called IS*1595 *[13]. Transposases were among the largest due to relatively long N3-C1 spacers, which were sometimes twice as long as those from family IS*4*. Only five out of 32 displayed the arginine residue of the YREK motif (results not shown, see Figure 2). This large group could be divided in (three) distinct clusters according to transposases (Additional file 10). Target site duplications were five to six bp AT-rich tracts while DNA ends were only poorly conserved. They were remarkably diverse in archaea and relatively widespread in bacteria (Figure 4).

### Distribution of copy numbers

**Figure 5:**
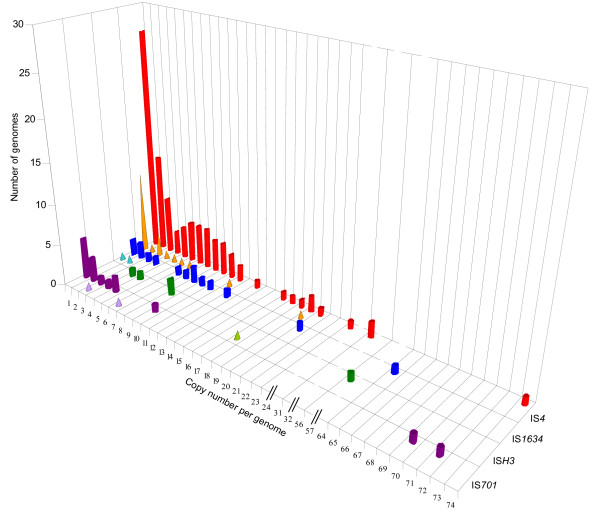
**Genomic IS copy number**. Genomic copy numbers of four families as they were found on chromosomes and plasmids. Families IS*4*, IS*1634*, IS*H3 *and IS*701 *are represented in red, blue, green and purple, respectively. The number of copies located on chromosomes is represented by cylinders while those located on plasmids is shown as cones. The height of each cylinder/cone indicates the number of chromosome/plasmid displaying the corresponding number of IS family members. No distinction was made when different elements of the same IS family occurred in the same genome. The histograms include intact elements, disrupted full length copies and large partial fragments displaying more than 95 % DNA sequence identity. Genomes without any copy of the aforementioned families were not included.

Members of the IS*4 *family were found in 92 out of 540 (~17 %) complete genomes representing 65 prokaryotic species. Families IS*701*, IS*H3 *and IS*1634 *displayed lower incidences, being present in ~3 %, ~0.7 % and ~2 % of available genomes, respectively. 172 of these genomes included plasmids of which 22 harbored elements of these families. Figure 5 displays the distribution of IS genomic copy numbers as it was found on chromosomes and plasmids. The distribution of family IS*4 *uncovered a clear preference for single genomic copies, followed by a preference for two, three and six to eight copies per genome. The genomes of *Photobacterium profundum *SS9, *Mycoplasma mycoides *subsp. *mycoides *SC strain PG1, *Sulfolobus solfataricus *P2 and the two sequenced strains of *Xanthomonas oryzae *pv. *oryzae *displayed the highest amounts of elements from families IS*4*, IS*1634*, IS*H3 *and IS*701*, respectively (Figure 5). The most IS-rich extrachromosomal replicons were megaplasmids pNRC100 (191 kb) and pNRC200 (365 kb) from *Halobacterium sp*. NRC-1, which harbored together 20 copies of IS*4 *family elements and 18 copies of family IS*H3 *elements.

### Distribution of IS subgroups and families among prokaryotic phyla and viruses

The distribution of individual elements among the different bacterial and archaeal phyla is shown in Figure 4. As indicated by the histogram in Figure 4B, current sequencing projects focus unequally on different prokaryotic phyla [27]. The distribution presented in Figure 4A partially reflected this sequencing bias, which is why most gathered elements were found in proteobacteria. Globally, families IS*4*, IS*701*, IS*H3 *and IS*1634 *were detected in eight, seven, two and seven phyla, respectively.

Figure 4A shows that the distribution of individual IS families or subgroups as defined in this study could either be broad (as for subgroups IS*4*, IS*4Sa *and family IS*701*) or concentrated in a particular phylum (as for subgroup IS*231 *and family IS*H3*). Interestingly, two copies of an element belonging to subgroup IS*4 *were found in the genome of the brown algae virus, *Ectocarpus siliculosus virus 1 *[28]. So far, no IS*4*-related element was found in eukaryotes.

## Discussion

### Impact of IS*4 *and related families on prokaryotic genomes

The availability of a large number of completely sequenced genomes on one hand, and the extensive manual characterization of an IS family on the other hand, allowed a direct evaluation of global presence of its putatively functional members. Family IS*4 *displayed a sporadic distribution among 17 % of explored genomes. Moreover, the behavior of these ISs was assessed by determining their genomic occurrence. This showed that numerous elements exhibited single or low copy numbers, which was in line with the assumption that transposable elements need to compromise between transposition for self maintenance and limited insertions to keep host genome integrity [29]. This distribution also displayed a certain, less pronounced, prevalence for copy numbers between 6 and 8, although the biological relevance of this observation remains unclear.

While the global distribution of IS elements was shown to be rather sporadic, important expansion events were observed in a few pathogens as well as in some free-living extremophiles. One of these pathogens is a member of the facultative intracellular *Mycoplasma*, of which eleven genomes are available. 65 intact copies of family IS*1634 *elements were found together with 17 intact copies of the IS*3 *family in *M. mycoides *[30]. The presence of family IS*1634 *was also detected in *M. synoviae *[31], *M. agalactiae *[GenBank:CU179680] and the three strains of *M. hyopneumoniae *[31,32]. Only *M. genitalium *[33], *M. pneumoniae *[34] and *M. mobile *[35] were devoid of ISs. Interestingly, the *Mycoplasma *species lacking ISs were also those displaying the smallest genomes (between 0,58 and 0,82 Mb), while the IS-rich *M. mycoides *had a relatively large genome (1,2 Mbs). A positive correlation between the total number of genes and the amount of mobile DNA in a genome has been reported for other facultative intracellular bacteria [36], and a recent study has extended this observation to other prokaryotes [37]. However, this relationship must be seen as a trend with notable exceptions (e.g. the large genome of *M. penetrans *– 1,36 Mbs – has only 21 transposase genes [38]).

Family IS*701 *was represented by 70 and 72 elements in the genomes of two sequenced pathovars of the rice pathogen *X. oryzae *[39,40]. This family was completely absent in phylogenetically related species with available genome sequences, including *X. campestris *(black rot in crucifers) and *X. axonopodis *(citrus canker) [39-43]. Additionally, a moderate presence of family IS*4 *was observed in all but one of the six *Xanthomonas *genomes (*X. campestris *pv. *vesicatoria*), together with other, more expanded families. So far, all sequenced xanthomonads are IS-rich, which is supposed to provide the necessary flexibility for adaptation to different plant hosts [40]. Interestingly, each strain is characterized by the expansion of one specific IS family [43]. The IS*4 *family was also observed to be relatively expanded in sequenced *Shigella *spp. where other families, both with higher and lower expansion levels, were also detected [44-47]. These mobile elements are supposed to contribute to the emergence of variable epidemiological and pathological features among these phylogenetically close pathogens [45].

These observations are reminiscent of IS expansions observed together with emergence of pathogenicity in other host associated pathogens, such as *Bordetella pertussis *(whooping cough [48]), *Yersinia pestis *(plague [49]) and *Francisella tularensis *(tularemia [50]).

A different relationship was observed in the genus *Vibrio*. The expansion of family IS*4 *in the non-pathogenic *Photobacterium *[51] was striking (Figure 5) as compared to those observed in the pathogenic *Vibrionaceae*, such as *V. cholerae *[52], *V. parahaemolyticus *[53] and *V. vulnificus *[54], that displayed relatively modest IS contents. Therefore, ISs apparently have a quite different role in *Vibrio *evolution, where some IS-rich species are non-pathogenic piezophiles. Other important IS expansions among free-living extremophiles were observed in one out of three *Sulfolobus *spp. [55-57], where family IS*H3 *represents a significant fraction of global IS content, as well as in *Halobacterium*, which harbors numerous copies of IS*4 *and IS*H3 *elements together with other families [58].

### Lateral transfer and clade specificity

Analysis of the distribution of individual elements among prokaryotes and among established families and subgroups was also performed regardless of copy numbers (Figure 4). Prokaryotic IS hosts were from world-wide origins and covered a large range of lifestyles and habitats, from free-living environmental germs and extremophiles to host associated symbionts and pathogens. Evaluation of emergence on evolutionary timescales, as well as estimation of horizontal gene transfer (HGT) extent prompted by this distribution remain both problematic because of low identity levels among transposases. However, many IS*4*-related elements were found on putative mobile DNA, including various plasmids (Figure 5), bacteriophages (results not shown) and one eukaryotic virus (Figure 4). Additionally, most IS*4*-related subgroups and emerging families were scattered over several phyla (Additional files 1, 2, 3, 4, 5, 6, 7, 8, 9, 10), suggesting an involvement of HGT in the dissemination of these elements.

Reports on prokaryotic IS distribution have shown that IS families are not clade-specific and our data regarding family IS*4 *were in agreement with this assumption. However, the distribution of IS*4 *subgroups and emerging families showed that the number of elements of some IS groups was not systematically proportional to the extent of genome sequencing (Figure 4). Subgroup IS*231 *(family IS*4*) was almost exclusively restricted to Firmicutes and 26 of its 32 elements were found among bacteria of the *B. cereus s.l*. group. Likewise, family IS*1634*, and to a lesser extent family IS*H3 *and subgroup IS*H8*, were preferentially found among *Euryarchaeota*. These observations may partially be due to insufficient genome data, but they do not rule out the possibility of existing clade specificity at the level of IS subgroups. Preferential IS occurrences among certain prokaryotic phyla or genera can be the consequence of isolated niches reducing or limiting horizontal transfer of hosted ISs. In this case, a series of extremophilic archaea were almost exclusive hosts of subgroup IS*H8 *and family IS*H3*, their preference for harsh ecological niches may represent a frontier for HGT with other phyla or with bacteria. Another possible explanation can be based on IS-associated features. It is possible that the presence of some ISs cannot be tolerated by certain hosts. Uncontrollable transposition behaviors, lack of target site specificity, preferred insertions into vital genes or regulatory regions can limit the host compatibility of some ISs for which a viable equilibrium can be found only in rare 'IS – host' combinations.

### Reaction mechanisms

The co-crystal structure of the IS*50R *transposase binding the ends of the Tn*5 *transposon generated function assignments for numerous residues of this protein [9]. A comparative sequence analysis based on 19 transposases was performed and compared with a previous report on seven transposases from subgroup IS*50 *by Reznikoff *et al*. in 2004 [59]. This confirmed the previous function assignment of conserved residues since most identified domains were still conserved among this subgroup, as were a series of residues with unknown function (further results available in Additional file 11).

The primary sequence data concerning the transposase motifs (Figure 2) and target site duplications (Figure 3) suggested divergent biochemical behaviors between IS*4 *transposases and those from the emerging families. The two 3'-OH transposon ends are known to attack the target helix at opposite sites in the case of IS*4*. For the emerging families, the length of target site duplications (5 bp) suggested a different target strand cleavage path: owing to the structure of B-DNA, this attack needs to come from the same helix side. It is worth noting that in the case of family IS*1634*, two elements were reported to display atypical target site duplication lengths. IS*1549 *and IS*1634*, from *M. smegmatis *and *M. mycoides*, respectively, have both been shown to produce long, variable-length DRs upon insertion [60,61]. They had lengths between 8 and 514 bps, depending on the copy. So far, no mechanistic model has been proposed for the generation of this variable target duplication size.

## Conclusion

This study consisted in a thorough and systematic screen for IS*4*-related elements among available genome sequence data. It allowed a considerable improvement regarding the description and definition of family IS*4*, as well as the establishment of new IS families which were, until now, assimilated to the IS*4 *ensemble (Table 1). Thus, 22 distinct IS families are now officially established. Novel primary sequence descriptions were unraveled for reported families, which will allow easier identification of other related elements as they will be uncovered with ongoing genome sequencing projects. This work also established a link between genomic and functional data regarding reaction mechanisms, which underlined the importance of both approaches for a more complete understanding of transposition biology.

The extent of genomic impact of single IS families showed that IS*4 *and its related families are far from being ubiquitous among prokaryotes. This limited IS distribution applies to most known IS families as reported by a recent automated survey of bacterial ISs [62]. Significant genome wide expansions were observed only in a few host-associated pathogens and certain free-living extremophiles, suggesting that particular ISs could have been, at least partially, implicated in the emergence or evolution of these particular lifestyles. Yet, the reasons explaining these sporadic IS demographic explosions remain to be uncovered.

Our interpretation of these results is that the IS families described here, and probably ISs in general, represent an evolutionary tool available among several. In order to provide the necessary genome flexibility for adaptation to new or variable environments, evolution seems to select this tool in some cases. In contrast to a purely 'selfish' or 'parasitic' perception of mobile DNA, this view supports the idea that prokaryotes, and maybe life in general, may also make use of mobile DNA for its own benefits, rather than being constantly invaded by it in an uncontrollable manner.

## Methods

### Genomic *in silico *screen for IS*4*-like elements

This search covered more than 500 complete and partial bacterial genomes. Only apparently full-length transposases were retained to avoid inclusion of partially deleted and thus inactivated transposases which can accumulate mutations of functionally important residues.

When we began our search for novel IS*4*-like elements, the ISfinder database contained about 70 ISs designated as 'member of family IS*4*'. First, representative elements (about twenty) covering most of the sequence diversity of this family were selected. Primary transposase sequence of each of these representatives was then used in a BLASTP search, either among microbial genomes only, or against all organisms. Since IS*4 *elements exhibit short TIRs and generate target site duplications upon insertion (DRs), the flanking DNA of resulting hits was checked for the presence of these repeats. This, together with DNA extremity comparison of various elements, allowed the determination of both ends of the collected elements. New ISs were checked for the existence of formerly registered iso-forms which we defined as elements with less than 2% divergence in the amino acid sequence of their putative transposases and/or less than 5% difference in their DNA sequences. ISs were submitted to the ISfinder database, which provided new names according to the current IS nomenclature [15].

### Bioinformatic procedures

BLASTP searches were performed on the NCBI BLAST online interface [27] without low complexity filter and with otherwise default parameters. Each transposase sequence retrieved a series of protein hits which were possible transposase candidates. The DNA encoding these candidates was downloaded together with 1000 bps up- and downstream regions. These DNA sequences were then verified for the presence of TIRs flanking the transposase candidate genes using BLASTN and the PALINDROME algorithm of the wEMBOSS package at the Belgian EMBnet Node [63]. If TIRs were present, flanking DRs were looked for by eye.

The following multiple alignment algorithms were then evaluated with the resulting ISs for their performance to accurately align the catalytic D, D and E residues of transposases : Clustal W [64], Dialign [65], Parallel PRRN [66], Muscle [67], T-Coffee [68] and M-Coffee [69]. Global alignment of the 227 transposases was made by merging existing M-Coffee alignments with Clustal W. The following order was applied where pre-existing alignments are represented by subgroup or family designations flanked by brackets and merging procedures by + symbols: (((IS*231*)+(IS*H8*)+(IS*4Sa*)+(IS*4*))+(IS*10*, IS*50*, IS*Pepr1*))+((IS*701*, IS*H3*)+(IS*1634*)). Examination of resulting alignments and subgroup specific dendrogram construction (UPGMA, BLOSUM62) were performed using the Jalview alignment editor [70]. Dendrograms were drawn with TreeView [71].

In order to facilitate visualization of transposase clusters, the TRIBE-MCL clustering algorithm [72] was applied to the complete set of transposases with inflation option (-I) set to 1,2 and default values for other parameters. These settings corresponded to those used by the ISfinder [15] and ACLAME [73] databases.

### Evaluation of IS impact on sequenced genomes

587 chromosomes and 363 plasmids, representing 540 individual completely sequenced bacterial and archaeal genomes, were screened for the presence of IS*4*, IS*701*, IS*H3 *and IS*1634 *family DNA. Therefore, the 950 molecules were used as input in BLASTN [74] searches against a homemade database containing the 227 DNA sequences encompassing these IS families.

## Authors' contributions

DDP carried out the genomic *in silico *screen for IS*4*-related elements, aligned DNA and protein sequences of gathered elements, collected insertion sites, proposed the present classification, submitted new elements to the ISfinder database and drafted the manuscript. PS conceived of the IS retrieval strategy, carried out the Tribe-MCL clustering, participated in analysis and interpretation of data and helped to draft the manuscript. JM participated in design of study and its coordination, participated in interpretation of data and critically revised the manuscript several times. All authors read and approved the final manuscript.

## Supplementary Material

Additional file 1 Subgroup IS*231*. **A. **Dendrogram displaying relative distances of transposases from subgroup IS*231*. Each tree leave indicates the name of the associated element, followed by the host organism and prokaryotic phylum in which the IS was found originally. For a complete description of individual elements please refer to the ISfinder database [15]. **B. **and **C. **Alignment of left and right DNA extremities, respectively. Names of corresponding elements are listed in the same order as in A. The blue color scheme represents the percentage of nucleotide identity per column as displayed by black bars. The DNA extremity consensus used in Figure 1 is shown in bottom, together with minimal (black line) and maximal (dashed line) extent of TIRs. IRL, left TIR; IRR, right TIR. Click here for file

Additional file 2 Subgroup IS*H8*. **A. **Dendrogram displaying relative distances of transposases from subgroup IS*H8*. Each tree leave indicates the name of the associated element, followed by the host organism and prokaryotic phylum in which the IS was found originally. For a complete description of individual elements please refer to the ISfinder database [15]. **B. **and **C. **Alignment of left and right DNA extremities, respectively. Names of corresponding elements are listed in the same order as in A. The blue color scheme represents the percentage of nucleotide identity per column as displayed by black bars. The DNA extremity consensus used in Figure 1 is shown in bottom, together with minimal (black line) and maximal (dashed line) extent of TIRs. IRL, left TIR; IRR, right TIR. Click here for file

Additional file 3 Subgroup IS*4Sa*. **A. **Dendrogram displaying relative distances of transposases from subgroup IS*4Sa*. Each tree leave indicates the name of the associated element, followed by the host organism and prokaryotic phylum in which the IS was found originally. For a complete description of individual elements please refer to the ISfinder database [15]. **B. **and **C. **Alignment of left and right DNA extremities, respectively. Names of corresponding elements are listed in the same order as in A. The blue color scheme represents the percentage of nucleotide identity per column as displayed by black bars. The DNA extremity consensus used in Figure 1 is shown in bottom, together with minimal (black line) and maximal (dashed line) extent of TIRs. IRL, left TIR; IRR, right TIR. Click here for file

Additional file 4 Subgroup IS*4*. **A. **Dendrogram displaying relative distances of transposases from subgroup IS*4*. Each tree leave indicates the name of the associated element, followed by the host organism and prokaryotic phylum in which the IS was found originally. For a complete description of individual elements please refer to the ISfinder database [15]. **B. **and **C. **Alignment of left and right DNA extremities, respectively. Names of corresponding elements are listed in the same order as in A. The blue color scheme represents the percentage of nucleotide identity per column as displayed by black bars. The DNA extremity consensus used in Figure 1 is shown in bottom, together with minimal (black line) and maximal (dashed line) extent of TIRs. IRL, left TIR; IRR, right TIR. Click here for file

Additional file 5 Subgroup IS*10*. **A. **Dendrogram displaying relative distances of transposases from subgroup IS*10*. Each tree leave indicates the name of the associated element, followed by the host organism and prokaryotic phylum in which the IS was found originally. For a complete description of individual elements please refer to the ISfinder database [15]. **B. **and **C. **Alignment of left and right DNA extremities, respectively. Names of corresponding elements are listed in the same order as in A. The blue color scheme represents the percentage of nucleotide identity per column as displayed by black bars. The DNA extremity consensus used in Figure 1 is shown in bottom, together with minimal (black line) and maximal (dashed line) extent of TIRs. IRL, left TIR; IRR, right TIR. Click here for file

Additional file 6 Subgroup IS*50*. **A. **Dendrogram displaying relative distances of transposases from subgroup IS*50*. Each tree leave indicates the name of the associated element, followed by the host organism and prokaryotic phylum in which the IS was found originally. For a complete description of individual elements please refer to the ISfinder database [15]. **B. **and **C. **Alignment of left and right DNA extremities, respectively. Names of corresponding elements are listed in the same order as in A. The blue color scheme represents the percentage of nucleotide identity per column as displayed by black bars. The DNA extremity consensus used in Figure 1 is shown in bottom, together with minimal (black line) and maximal (dashed line) extent of TIRs. IRL, left TIR; IRR, right TIR. Click here for file

Additional file 7 Subgroup IS*Pepr1*. **A. **Dendrogram displaying relative distances of transposases from subgroup IS*Pepr1*. Each tree leave indicates the name of the associated element, followed by the host organism and prokaryotic phylum in which the IS was found originally. For a complete description of individual elements please refer to the ISfinder database [15]. **B. **and **C. **Alignment of left and right DNA extremities, respectively. Names of corresponding elements are listed in the same order as in A. The blue color scheme represents the percentage of nucleotide identity per column as displayed by black bars. The DNA extremity consensus used in Figure 1 is shown in bottom, together with minimal (black line) and maximal (dashed line) extent of TIRs. IRL, left TIR; IRR, right TIR. Click here for file

Additional file 8 Family IS*701*. **A. **Dendrogram displaying relative distances of transposases from family IS*701*. Each tree leave indicates the name of the associated element, followed by the host organism and prokaryotic phylum in which the IS was found originally. For a complete description of individual elements please refer to the ISfinder database [15]. **B. **and **C. **Alignment of left and right DNA extremities, respectively. Three distinct alignments, corresponding to three different DNA end signatures, are shown in this case. Names of corresponding elements are listed in the same order as in A. Blue color scheme represents percentage of nucleotide identity per column in each alignment, and is represented by black bars for the upper alignment only. Minimal (black line) and maximal (dashed line) extent of TIRs is given in each case. IRL, left TIR; IRR, right TIR. Click here for file

Additional file 9 Family IS*H3*. **A. **Dendrogram displaying relative distances of transposases from family IS*H3*. Each tree leave indicates the name of the associated element, followed by the host organism and prokaryotic phylum in which the IS was found originally. For a complete description of individual elements please refer to the ISfinder database [15]. **B. **and **C. **Alignment of left and right DNA extremities, respectively. Names of corresponding elements are listed in the same order as in A. The blue color scheme represents the percentage of nucleotide identity per column as displayed by black bars. The DNA extremity consensus used in Figure 1 is shown in bottom, together with minimal (black line) and maximal (dashed line) extent of TIRs. IRL, left TIR; IRR, right TIR. Click here for file

Additional file 10 Family IS*1634*. **A. **Dendrogram displaying relative distances of transposases from family IS*1634*. Each tree leave indicates the name of the associated element, followed by the host organism and prokaryotic phylum in which the IS was found originally. For a complete description of individual elements please refer to the ISfinder database [15]. **B. **and **C. **Alignment of left and right DNA extremities, respectively. Names of corresponding elements are listed in the same order as in A. The blue color scheme represents the percentage of nucleotide identity per column as displayed by black bars. The DNA extremity consensus used in Figure 1 is shown in bottom, together with minimal (black line) and maximal (dashed line) extent of TIRs. IRL, left TIR; IRR, right TIR. Click here for file

Additional file 11 Combining alignment data from subgroup IS*50 *with functional data from transposons Tn*5*. The black sequence is the consensus obtained after multiple alignments of 19 members of the IS*50 *subgroup with MCOFFEE. Interrupting dashes indicate alignment gaps. The conservation percentage is represented by black bars over each position. The dotted line stands for 70% residue conservation. Residues of the IS*50R *transposase with known functional data are shown together with their position coordinates. They are grouped and color-coded following the function they carry out in regulation or during the transposition mechanism. Aspartate and glutamate residues of the DDE motif are pointed out by white triangles among the conservation bars. The YREK motif is highlighted by white 'V'. The transpososome formation step (i) is divided into successive stages including initial *cis*-binding, dimerization, 3'-end & 5'-end *trans *binding, *trans*-binding via the *β *hairpin clamp and additional *trans *contacts. Target capture residues are displayed by (ii), while integration and transposase release are not shown. Residues annotated as intramolecular inhibition are supposed to inhibit dimerization of full length transposases. For further details about these mechanistic concepts, see references [9,25,59]. Each residue is linked by colored lines to the corresponding position in the alignment consensus. Their designation is colored and bold if the residue is conserved at least at 70%, gray if it is less or not conserved. Conserved positions with unknown function are pointed out by asterisks (*). Click here for file
