# Supplementary material for: IS4 family goes genomic
Source: BMC Evol Biol. 2008 Jan 23;8:18. doi: 10.1186/1471-2148-8-18 (PMC2266710; doi:10.1186/1471-2148-8-18)

Additional file 4 :

Subgroup IS4

A. Transposase based dendrogram

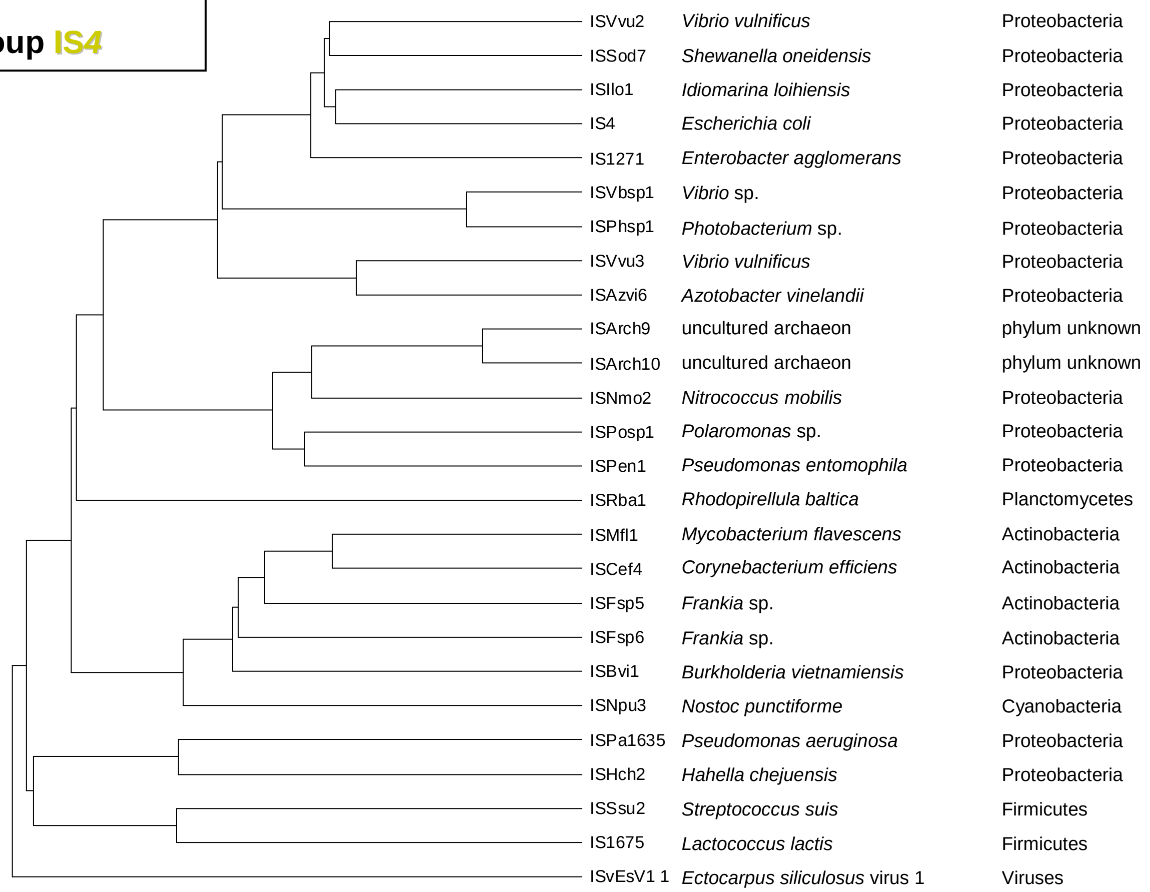

B. Left extremities

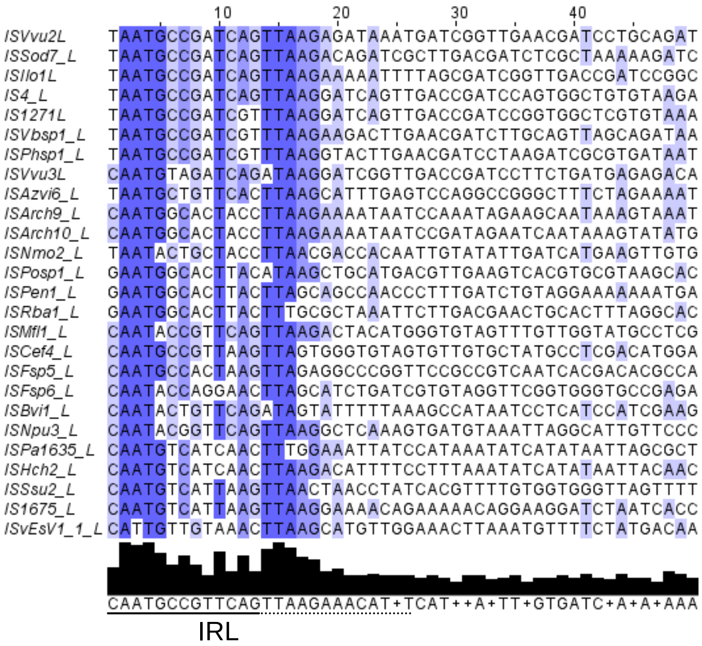

C. Right extremities

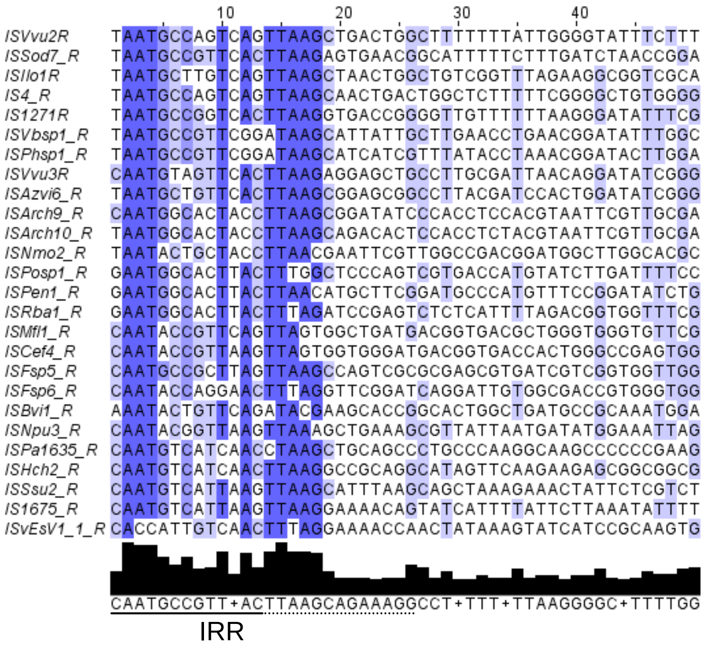

Supplement: Additional file 4 — Subgroup IS4. A. Dendrogram displaying relative distances of transposases from subgroup IS4. Each tree leave indicates the name of the associated element, followed by the host organism and prokaryotic phylum in which the IS was found originally. For a complete description of individual elements please refer to the ISfinder database [15]. B. and C. Alignment of left and right DNA extremities, respectively. Names of corresponding elements are listed in the same order as in A. The blue color scheme represents the percentage of nucleotide identity per column as displayed by black bars. The DNA extremity consensus used in Figure 1 is shown in bottom, together with minimal (black line) and maximal (dashed line) extent of TIRs. IRL, left TIR; IRR, right TIR. [file 1471-2148-8-18-S4.pdf]
