# Supplementary material for: IS4 family goes genomic
Source: BMC Evol Biol. 2008 Jan 23;8:18. doi: 10.1186/1471-2148-8-18 (PMC2266710; doi:10.1186/1471-2148-8-18)

Additional file 8 :

Family *IS701*

A. Transposase based dendrogram

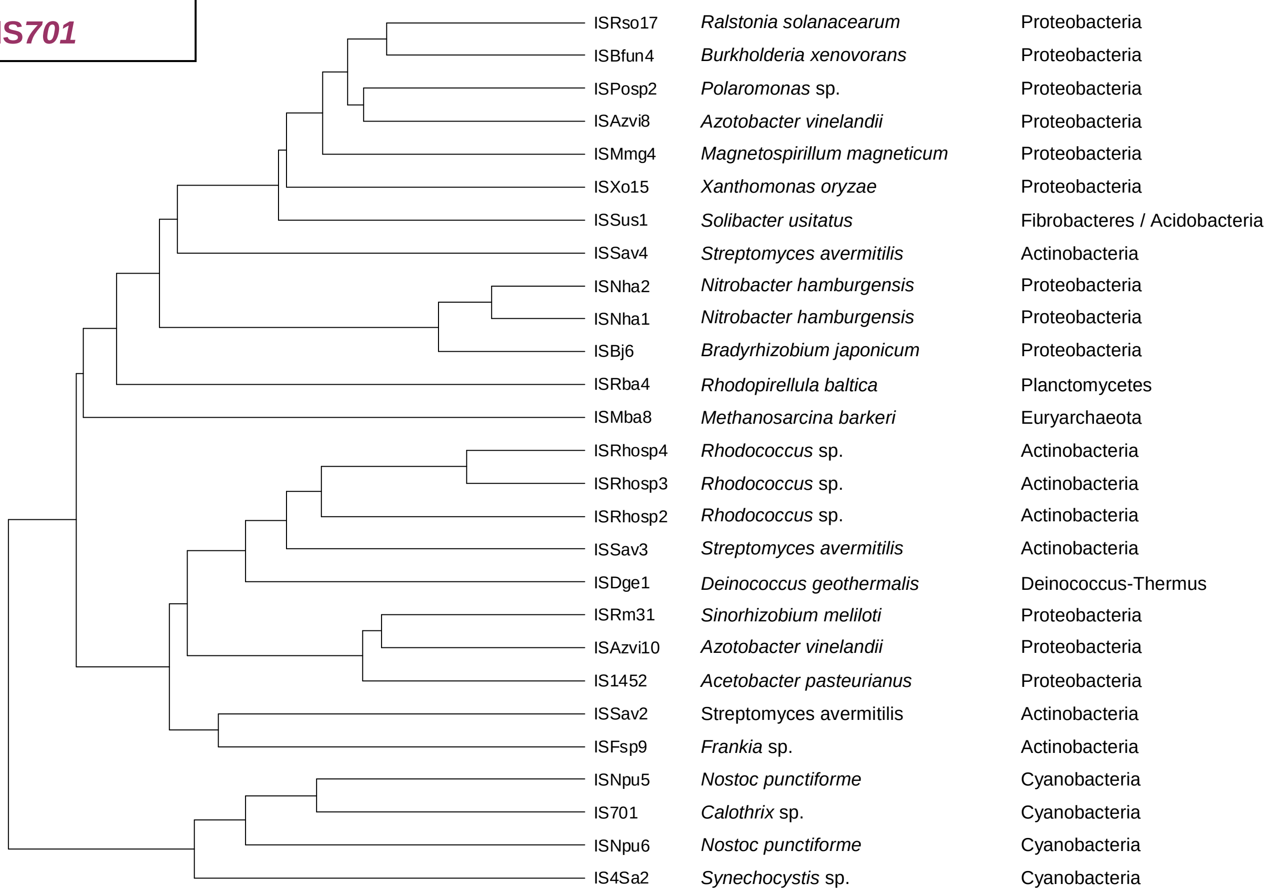

B. Left extremities

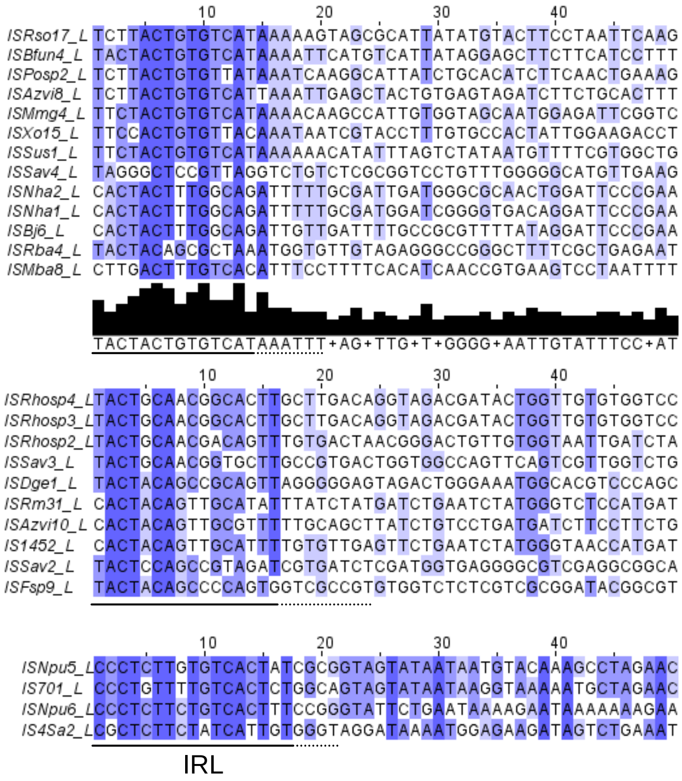

C. Right extremities

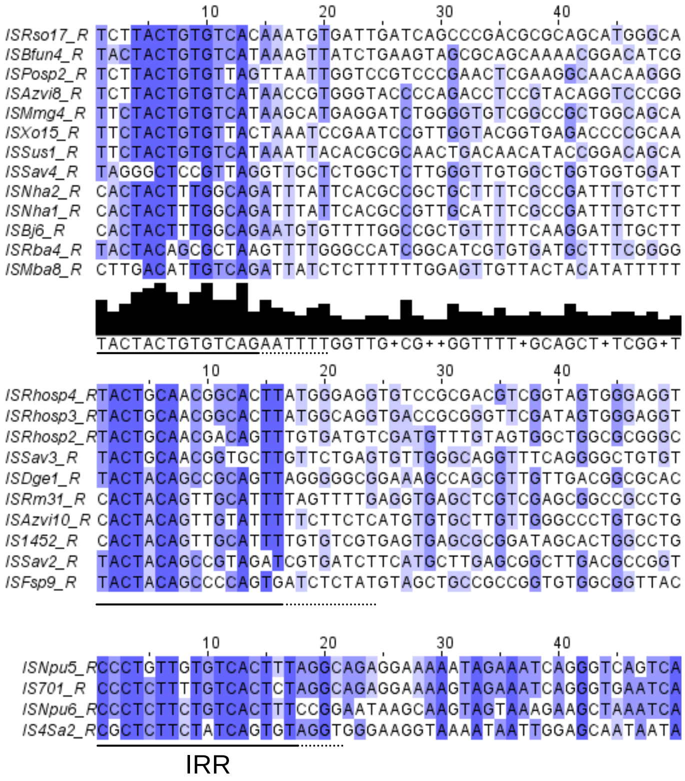

Supplement: Additional file 8 — Family IS701. A. Dendrogram displaying relative distances of transposases from family IS701. Each tree leave indicates the name of the associated element, followed by the host organism and prokaryotic phylum in which the IS was found originally. For a complete description of individual elements please refer to the ISfinder database [15]. B. and C. Alignment of left and right DNA extremities, respectively. Three distinct alignments, corresponding to three different DNA end signatures, are shown in this case. Names of corresponding elements are listed in the same order as in A. Blue color scheme represents percentage of nucleotide identity per column in each alignment, and is represented by black bars for the upper alignment only. Minimal (black line) and maximal (dashed line) extent of TIRs is given in each case. IRL, left TIR; IRR, right TIR. [file 1471-2148-8-18-S8.pdf]
