# Supplementary figures and images for: IS4 family goes genomic
Source: BMC Evol Biol. 2008 Jan 23;8:18. doi: 10.1186/1471-2148-8-18 (PMC2266710; doi:10.1186/1471-2148-8-18)

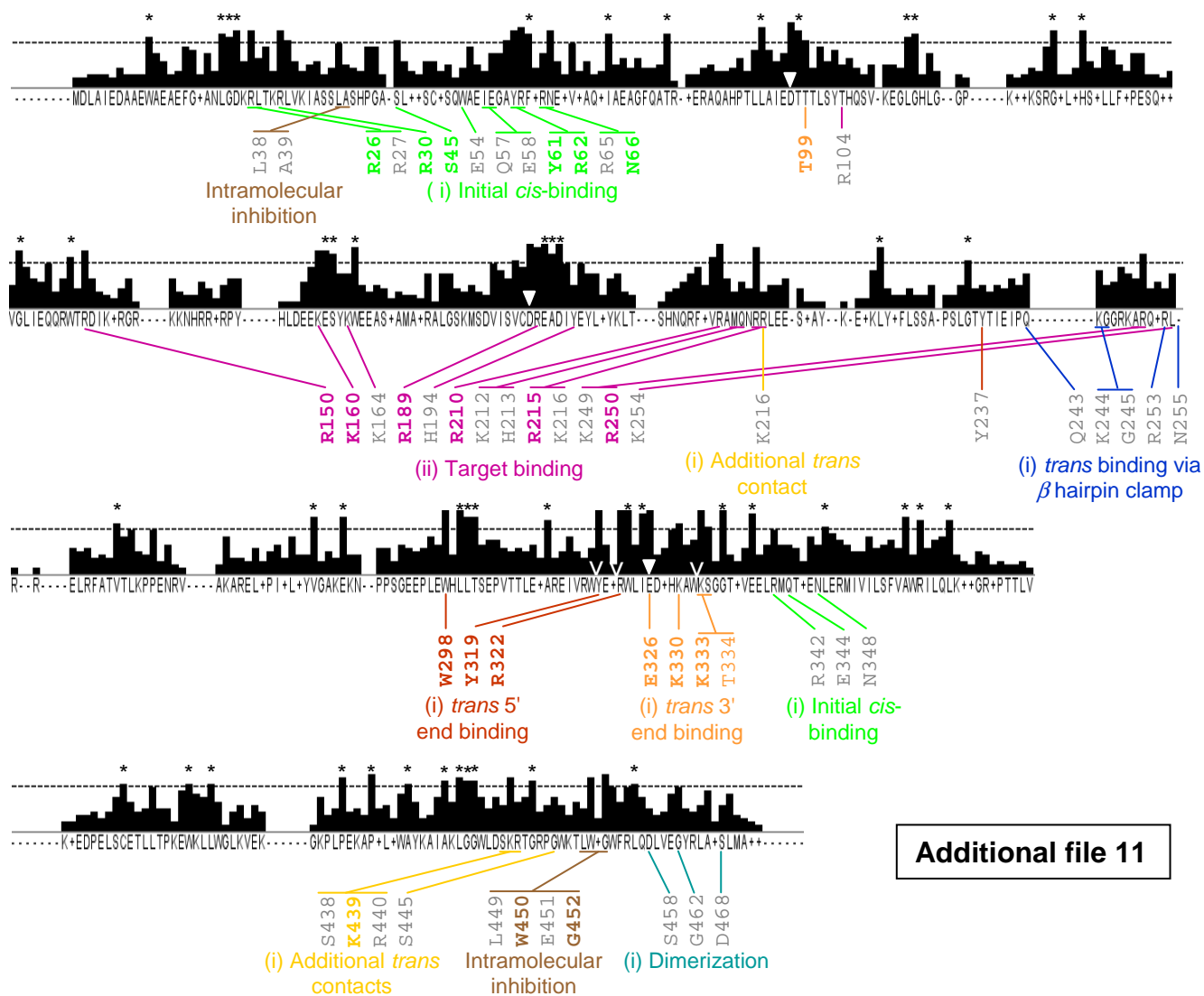

Additional file 11

Supplement: Additional file 11 — Combining alignment data from subgroup IS50 with functional data from transposons Tn5. The black sequence is the consensus obtained after multiple alignments of 19 members of the IS50 subgroup with MCOFFEE. Interrupting dashes indicate alignment gaps. The conservation percentage is represented by black bars over each position. The dotted line stands for 70% residue conservation. Residues of the IS50R transposase with known functional data are shown together with their position coordinates. They are grouped and color-coded following the function they carry out in regulation or during the transposition mechanism. Aspartate and glutamate residues of the DDE motif are pointed out by white triangles among the conservation bars. The YREK motif is highlighted by white 'V'. The transpososome formation step (i) is divided into successive stages including initial cis-binding, dimerization, 3'-end & 5'-end trans binding, trans-binding via the β hairpin clamp and additional trans contacts. Target capture residues are displayed by (ii), while integration and transposase release are not shown. Residues annotated as intramolecular inhibition are supposed to inhibit dimerization of full length transposases. For further details about these mechanistic concepts, see references [9,25,59]. Each residue is linked by colored lines to the corresponding position in the alignment consensus. Their designation is colored and bold if the residue is conserved at least at 70%, gray if it is less or not conserved. Conserved positions with unknown function are pointed out by asterisks (*). [file 1471-2148-8-18-S11.pdf]
